# Quantification of tear glucose levels and their correlation with blood glucose levels in dogs

**DOI:** 10.1002/vms3.788

**Published:** 2022-03-19

**Authors:** Eunji Lee, Seonmi Kang, Jaeho Shim, Dajeong Jeong, Youngseok Jeong, Junyeong Ahn, Kangmoon Seo

**Affiliations:** ^1^ Department of Veterinary Clinical Sciences College of Veterinary Medicine and Research Institute for Veterinary Science Seoul National University Seoul Korea

**Keywords:** blood glucose, diabetes mellitus, dog, hyperglycaemia, tear collection, tear glucose

## Abstract

**Background:**

No previous studies have quantified tear glucose (TG) levels in dogs or compared changes in TG and blood glucose (BG) concentrations.

**Objective:**

To quantify TG concentration and evaluate its correlation with BG level in dogs.

**Methods:**

Twenty repetitive tests were performed in alternate eyes of four dogs, with a minimum washout period of 1 week. Tears and blood were collected at 30‐min intervals with successive glucose injections (1 g/kg) every 30 min. Cross‐correlations of BG and TG levels were assessed. The delay and association between TG and corresponding BG levels were analysed for each dog; samples were collected at 5‐min intervals. The tears were collected using microcapillary tubes. Collected tears and blood were analysed for glucose concentration using a colorimetric assay and commercially available glucometer, respectively.

**Results:**

The average baseline BG and TG levels were 4.76 ± 0.58 and 0.39 ± 0.04 mmol/L, respectively. Even with highly fluctuating BG levels, a significant cross‐correlation coefficient (*r* = 0.86, *p* < 0.05) was observed between changes of BG and TG levels. The delay time between BG and TG levels was 10 min. On average, BG levels were 16.34 times higher than TG levels. There was strong correlation between BG and TG levels (*r*_s_ = 0.80, *p* < 0.01). Significant differences in TG concentrations between normoglycaemia, mild hyperglycaemia, and severe hyperglycaemia were found (*p* < 0.05).

**Conclusions:**

Canine TG concentrations have not been quantified previously. Our findings suggest preliminary data for future research on TG levels in dogs and show TG measurement could be used to screen for diabetes mellitus in dogs.

## Graphical abstract

This study quantitatively measured tear glucose levels in dogs with experimentally induced hyperglycaemia. A significant cross‐correlation coefficient was observed between changes of blood glucose and tear glucose levels. The delay time between blood glucose and tear glucose levels was 10 min.

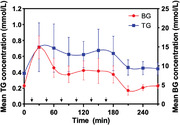

## 1 INTRODUCTION

Diabetes mellitus (DM) is a common endocrine disorder in dogs (Davison et al., 2005). Its prevalence in dogs (Heeley et al., 2020) was reported to be approximately 0.26%, and cases of canine diabetes have been rising worldwide, presumably as the result of changes in the lifestyles of dogs due to human urbanisation and preference for breeds predisposed to DM (Denyer et al., 2021, Kumar et al., 2014). Although it has been reported that dogs experience clinical symptoms 1.3 months prior to the diagnosis of DM on average, owners have difficulty recognising the non‐specific symptoms (Hess et al., 2000). Vision loss due to the rapid progression of cataracts is often observed before DM is diagnosed (Basher & Roberts, 1995). In total, 15% of dogs with DM presented with coexisting ketoacidosis (Hess et al., 2000). To prevent the life‐threatening complications of DM, including diabetic ketoacidosis, early diagnosis of DM as well as management of diabetic patients is crucial (Gilor et al., 2016).

Sampling of peripheral venous or capillary blood from the pinna or paw pad using a lancing device is a conventional method of measuring blood glucose (BG) in veterinary medicine (Ettinger et al., 2017). However, these methods are invasive and can cause pain and stress to animals (Davison et al., 2003). Recently, a continuous glucose monitoring device using interstitial fluid (ISF) has been introduced and used in veterinary clinical practice for glycaemic management, but not for screening for DM (Davison et al., 2003).

Alternatively, glucose screening using external body fluids that can be non‐invasively collected, such as tears, saliva, sweat, or urine, has been studied in humans (Baca et al., 2007; Moyer et al., 2012; Yamaguchi et al., 1998). Several previous studies have attempted to demonstrate the correlation between BG and tear glucose (TG) levels in humans (Baca et al., 2007) and to develop non‐invasive TG sensors, such as contact lenses (Badugu et al., 2004; Park et al., 2018) and flexible spring‐like devices placed under the lower eyelid (Kownacka et al., 2018).

However, there are only limited studies of the association between BG and TG in small animals (Cullen et al., 2005; Steinmetz et al., 2019). To date, only semi‐quantitative analysis of TG in dogs using a urine dipstick (Cullen et al., 2005) and a comparison of TG and BG concentrations in cats have been reported (Steinmetz et al., 2019). No studies have been conducted to quantify TG in dogs or to identify changes in TG in association with changes in BG.

The primary purpose of this study was to quantitatively evaluate TG levels in dogs and to identify the correlation between TG and BG levels. Therefore, we attempted to establish basic data for further studies assessing the feasibility of TG as a screening test for DM.

## 2 MATERIALS AND METHODS

### 2.1 Animals

Twenty tests were performed for evaluation of test reproducibility. The tests were performed in four male beagle dogs with a mean age of 15.75 ± 2.05 months (range: 14–19 months) and an average body weight of 11.68 ± 1.67 kg (10.0–13.6 kg). Prior to the study, ophthalmic examinations were performed including a neuro‐ophthalmic examination, Schirmer tear test (STT; Schirmer tear test strip; MSD Animal Health, Milton Keynes, UK), rebound tonometry (Tonovet^®^; Icare Finland Oy, Helsinki, Finland), slit‐lamp biomicroscopy (SL‐D7; Topcon, Tokyo, Japan), indirect ophthalmoscopy, and fluorescein staining (fluorescein paper; Hagg‐Streit AG, Koeniz, Switzerland). All dogs had normal STT values (>15 mm/min) and were confirmed to be free of ophthalmic diseases that could affect the composition of the tear film.

### 2.2 Study design

Prior to testing, food was withheld for 12 h; however, the animals were allowed free access to water. To minimise stress‐induced hyperglycaemia, the tests were carried out when the dogs were rested and calm.

Each test comprised a 5‐h course. Baseline fasting tear and blood samples were collected. Blood was withdrawn immediately after tear collection and performed as simultaneously as possible. Boluses of a glucose solution (1 g/kg) were administered through an intravenous catheter at 30‐min intervals for half of the total experiment duration (2 h 30 min). This repeated stimulation was designed to identify time‐course changes in the TG concentration following large fluctuations in the BG. The administered glucose dose was used for the intravenous glucose tolerance test (Bjornvad et al., 2014). One eye was randomly selected for the first test using a computerised method (Excel 2016; Microsoft Corp, Redmond, WA, USA). The following tests were conducted on alternate eyes. Each dog received a minimum washout period of 1 week between the experiments.

To determine the lag time between TG and BG levels in each dog, the difference between the time of each peak induced through a single glucose injection was measured. Tears and blood were collected at 5‐min intervals to identify the lag time between TG and BG elevation. A statistical analysis was performed for a total of 40 data points using a single glucose injection.

### 2.3 Tear and blood collection

Tears were collected by slightly everting the lateral canthus of the lower eyelid and carefully placing a 10‐μl microcapillary tube (Drummond Microcaps^®^; Drummond Scientific Co., Broomall, PA, USA) in the temporal inferior tear meniscus, while minimising contact with the conjunctiva and corneal surface. The capillary tube was removed after the 10‐μl microcapillary tube had completely filled with tears to obtain sufficient volume for tests. The sampling of this volume generally took less than 30 s, and it was performed as gently as possible to minimise reflex tearing. Spontaneous blinking was not inhibited. The tear sample within the microcapillary tube was immediately expelled into a 1.5‐ml Eppendorf tube (Eppendorf^®^ microtubes 3810X; Eppendorf AG, Hamburg, Germany). The tear samples in the Eppendorf tubes were centrifuged, and the tubes were immediately placed on ice and were frozen at –80℃ within 3 h of collection until further analysis. Blood samples were drawn from saphenous or cephalic veins immediately after tear sampling. After each experiment, fluorescein staining was performed on the tested eye to ensure no corneal damage had been caused by the microcapillary tube. Then, artificial tears (Refresh Plus^®^; Allergan Inc., Irvine, CA, USA) were administered.

### 2.4 Measurement of TG and BG levels

To measure the concentration of TG, a glucose colorimetric/fluorometric assay kit (MAK263; Sigma‐Aldrich Corp., St Louis, MO, USA) was used according to the manufacturer's instructions. Three microliters of tear sample were added to each well of a 96‐well plate, and all samples were run in duplicate. Absorbance was detected at 570 nm using a microplate spectrophotometer (Epoch microplate spectrophotometer; BioTek, Winooski, VT, USA). A commercially available portable glucometer (Accu‐Check^®^ Active; Roche Diagnostic Inc., Korea) was used to measure the concentration of BG from whole blood directly after sample collection.

### 2.5 Statistical analyses

Statistical analyses were performed using GraphPad Prism^®^ version 8.0 (GraphPad Software, San Diego, CA, USA) and SPSS version 25 (IBM Corp., Armonk, NY, USA). After evaluating normality of the data, a non‐parametric Wilcoxon signed‐rank test was performed to compare glucose concentrations between blood and tears. Cross‐correlation analysis was used for the two fluctuating time‐series changes. This allowed us to determine whether the two curves for mean BG and TG levels were correlated and when they were most relevant to each other. A linear regression analysis and Spearman rank correlation were performed to assess the association between BG concentration and the corresponding tear value. Additionally, BG concentrations were classified by the extent of hyperglycaemia as follows: normoglycaemia (4.44–6.66 mmol/L), hyperglycaemia (>6.66 mmol/L), mild hyperglycaemia (6.66–12.21 mmol/L), and severe hyperglycaemia (≥12.21 mmol/L). The criteria were based on veterinary internal medicine literature (Ettinger et al., 2017). Mild hyperglycaemia refers to an asymptomatic BG elevation that does not exceed the renal threshold for glucose reabsorption, and severe hyperglycaemia refers to significant hyperglycaemia that exceeds the renal tubular maximum for glucose reabsorption. The Mann–Whitney test was used to identify statistical differences in TG levels between each stage of BG concentration. A receiver operating characteristic (ROC) curve analysis was performed to evaluate the sensitivity and specificity in detecting hyperglycaemia.

Statistical significance was set at *p* < 0.05. All data are presented as the mean ± standard deviation.

## 3 RESULTS

### 3.1 Cross‐correlation between changes of BG and TG levels

**FIGURE 1:**
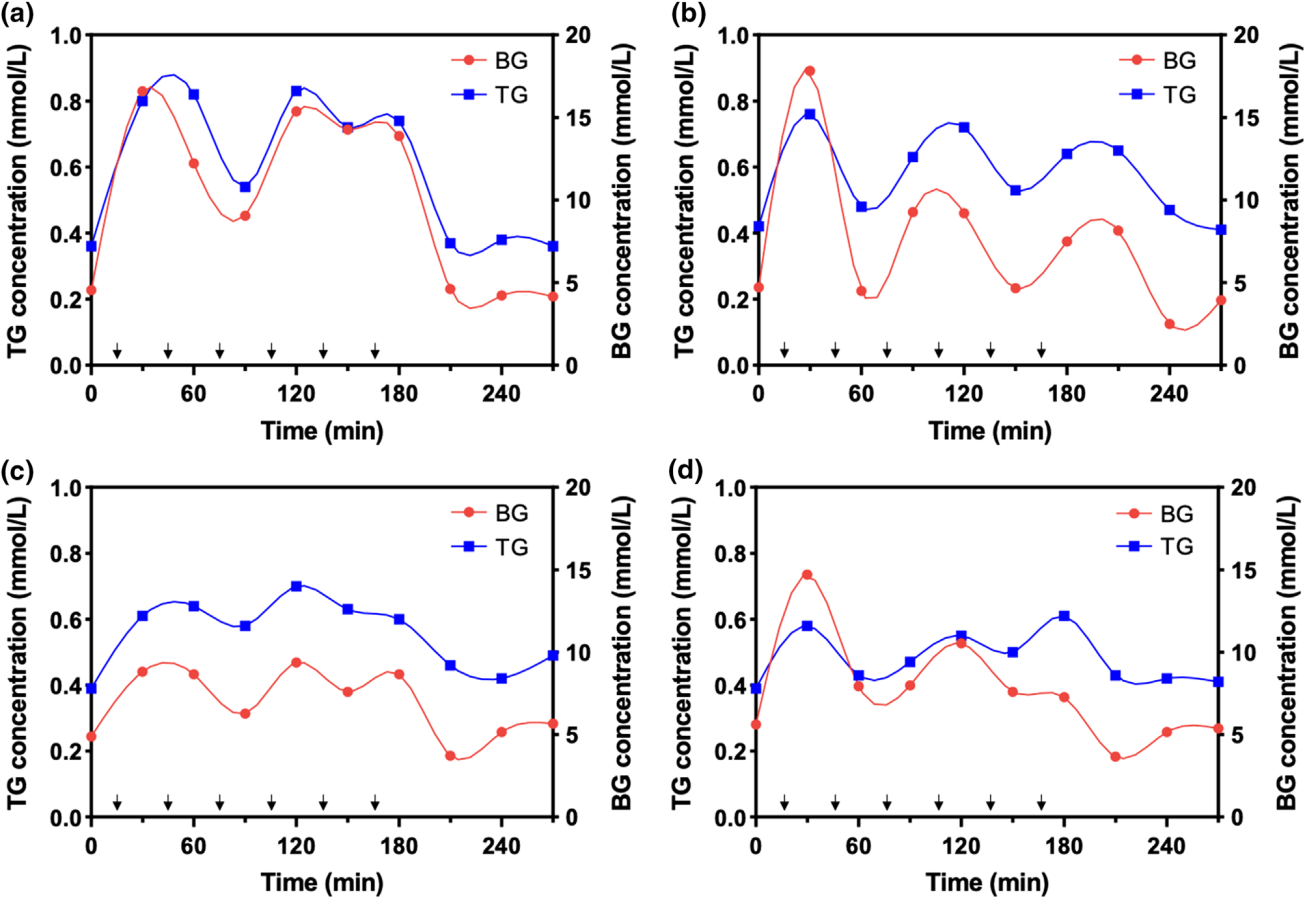
Time‐course changes in tear glucose (TG) and blood glucose (BG) concentrations with successive glucose injections in four dogs. The arrow represents the time point at which the glucose solution was administered. (a) Dog 1, (b) dog 2, (c) dog 3, and (d) dog 4

Repeated stimulation produced multiple peaks corresponding to hyperglycaemic, hypoglycaemic, and normoglycaemic ranges of BG. The time‐course changes of BG and TG levels for each dog were plotted (Figure 1). Prior to the first glucose solution administration, the mean baseline fasting BG level was 4.76 ± 0.58 mmol/L (range: 3.77–5.66 mmol/L), which was within the normal range. The mean TG level was 0.39 ± 0.04 mmol/L (range: 0.35–0.55 mmol/L). The BG concentrations were significantly higher than the TG concentrations (*p* < 0.01). Throughout the experimental courses, the values of BG and TG ranged from 2.28 to 21.31 and 0.34 to 1.73 mmol/L, respectively. The BG concentration increase was approximately ninefold, and the TG concentration increased fivefold. The TG changes followed fluctuating changes in BG in all individual tests.

**FIGURE 2:**
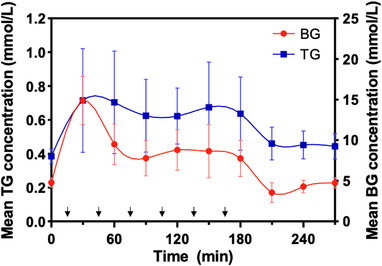
Time‐course changes in tear glucose (TG) and blood glucose (BG) concentrations with successive glucose injections in all dogs. Each dot represents the mean glucose concentration of 20 trials at the same time point. The arrow represents the time point at which the glucose solution was administered. The error bar represents the standard deviation. A significant cross‐correlation was observed at zero‐time lags between TG and BG levels in the cross‐correlation analysis (*r* = 0.86, *p* < 0.05)

Cross‐correlation analysis was performed to determine whether the two highly variable time‐series changes were correlated and where the best match occurred. For a total of 20 tests, a significant cross‐correlation (*r* = 0.86, *p* < 0.05) was identified between mean BG and TG levels at zero‐time lags (Figure 2). Since the experimental design allowed the collection of blood and tears at intervals of 30 min, this result indicates that the delay time for BG changes to be reflected in the tears would be between 0 and 30 min.

### 3.2 Lag time between BG and TG levels

**FIGURE 3:**
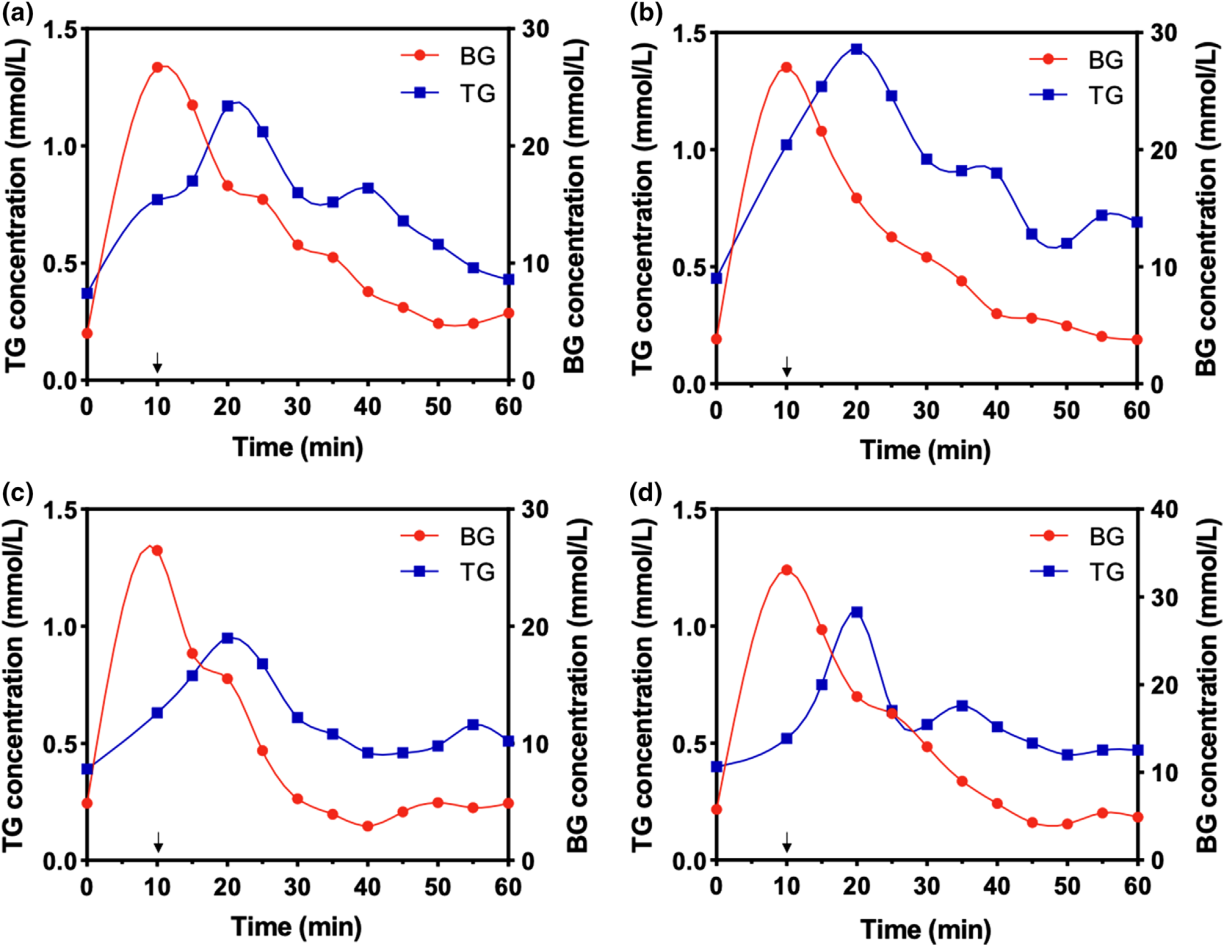
Chronological changes in tear glucose (TG) and blood glucose (BG) levels after single glucose injection in four dogs. The arrow represents the time point at which the glucose solution was administered. The peak of TG level was observed at a delay of 10 min from the peak of BG level. (a) Dog 1, (b) dog 2, (c) dog 3, and (d) dog 4

To determine the lag time between BG and TG level, tears and blood were collected at 5‐min intervals from each dog after a single glucose injection. After intravenous administration of glucose, BG levels increased immediately and reached the maximum level, while TG levels tracked BG levels in all subjects (Figure 3). However, the peak value for TG lagged 10 min behind that of BG in all dogs. The mean peak values of BG and TG were 28.32 ± 3.18 and 1.43 ± 0.21 mmol/L, respectively. The BG level decreased sequentially and returned to the normal range 30–35 min after injection. During the tests, the measured glucose values in blood and tears ranged from 2.94 to 33.08 mmol/L and from 0.37 to 1.43 mmol/L, respectively. The TG level increased by approximately four times, while the BG level increased by approximately 11 times.

### 3.3 Relationship between BG and corresponding TG levels

By synchronising the peaks of BG and TG by coinciding two peaks, the BG concentrations were paired with the corresponding TG values. Since the last two BG values had no corresponding TG values, they were excluded from the analysis used to calculate the correlation between BG and TG. The correlation analysis was performed for a total of 40 data points.

The TG concentration was significantly lower than the corresponding BG concentration (*p* < 0.01). The TG concentration was 7.52% of the corresponding average BG concentration (range: 2.44%–16.64%). The ratio of TG concentration to the corresponding BG concentration decreased as the BG level increased. The mean TG concentration was 9.01% of the BG concentration at baseline and 4.11% at the peak point.

**FIGURE 4:**
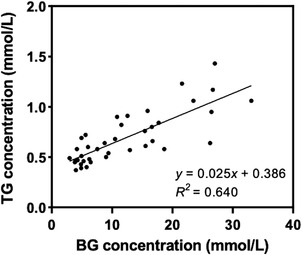
Scatter plot with linear regression demonstrating the relationship between tear glucose (TG) and blood glucose (BG) in all dogs. There was a strong correlation between TG and BG concentrations (*r*_s_ = 0.80, *p* < 0.01)

The relationship between BG levels and the corresponding TG levels is shown in Figure 4 with a linear regression line. A strong positive correlation was observed between the BG and TG concentrations in each dog (*p* < 0.05). The mean correlation coefficient (*r*_s_) in all dogs was 0.86 ± 0.11 (range: 0.71–0.96). There was also a strong correlation between BG and TG levels at all data points in all dogs (*r*_s_ = 0.80, *p* < 0.01) (Figure 4).

**FIGURE 5:**
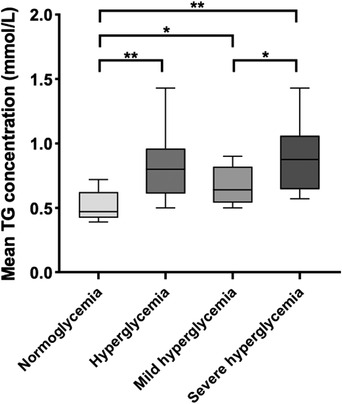
Box and whisker plots depicting the mean tear glucose (TG) concentration at different blood glucose (BG) stages. The median is represented by the horizontal bar, and the minimum and maximum values are shown as the lower and upper whiskers, respectively. Normoglycaemia is defined as BG between 4.44 and 6.66 mmol/L, hyperglycaemia as BG > 6.66 mmol/L, mild hyperglycaemia as BG between 6.66 and 12.21 mmol/L, and severe hyperglycaemia as BG ≥ 12.21 mmol/L. For each group evaluated, statistically significant differences are indicated by an asterisk (*p* < 0.05) and double asterisk (*p* < 0.01)

Next, we compared the TG levels at different BG levels. The mean TG level during hyperglycaemia was significantly higher than that during normoglycaemia (*p* < 0.01). Additionally, there was a significant difference between the mean TG levels during normoglycaemia and mild hyperglycaemia (*p* < 0.05). Furthermore, in the hyperglycaemic stage, there was a significant difference in TG concentrations between mild and severe hyperglycaemia (*p* < 0.05) (Figure 5). In other words, a distinct difference in TG concentration was observed between normoglycaemia, mild hyperglycaemia, and severe hyperglycaemia.

In the ROC curve analysis, TG concentrations ≥0.53 mmol/L had a sensitivity of 95.7% (95% confidence interval [CI]: 76.0%–99.8%) and a specificity of 76.5% (95% CI: 49.8%–92.2%) to detect hyperglycaemia (>6.66 mmol/L). The area under the curve was 92.3% (95% CI: 84.1%–100.0%).

## 4 DISCUSSION

The present study aimed to quantitatively measure TG levels in dogs with experimentally induced hyperglycaemia and to evaluate the statistical correlation between TG and BG levels. Our results showed a significant correlation between BG and TG levels. The glucose levels in blood and tears revealed a good match, showing the time difference in the experimentally induced fluctuation of BG. Additionally, a significant difference in TG levels was observed between normoglycaemia and hyperglycaemia.

The only previous study that measured TG concentrations in dogs documented median TG concentrations of 0 and 6 mmol/L in non‐diabetic and diabetic dogs, respectively (Cullen et al., 2005). Those results were similar to our findings in that there was a significant difference in TG concentrations between normal and hyperglycaemic groups. These findings were also consistent with the results of an earlier study that quantified TG in cats with normal or increased BG concentrations (Steinmetz et al., 2019). However, the TG concentrations in diabetic dogs (Cullen et al., 2005) (median: 6 mmol/L) and in hyperglycaemic cats (Steinmetz et al., 2019) (median: 1.50 mmol/L) were higher than those in the hyperglycaemic phase in the present study (median: 0.80 mmol/L). The possible reasons for these variations are as follows. First, the former study in dogs used a semi‐quantitative analysis with a urine dipstick; therefore, the value might be subjective with a rather wide measurement interval by comparing the colour of the reagent strip pad to a calibrated colour chart. Second, to collect the tears, Cullen et al. (2005) used a portion of the test stick placed in the meniscus at the medial canthus, and Steinmetz et al. (2019) placed polyurethane sponges in the ventral fornix, whereas a microcapillary tube for tear collection was used in the present study. Finally, the previous studies consisted of animals with disease such as DM and renal disease with hyperglycaemia, whereas this study induced transient hyperglycaemia in normal dogs. Therefore, it was difficult to directly compare the TG concentrations in the current study with those reported earlier.

Glucose is present in the aqueous layer of the tear film, and the aqueous layer is mainly secreted by the orbital lacrimal and nictitating membrane glands (Zhou & Beuerman, 2012). However, TG has a different mechanism, which is related to tissue fluid across the conjunctiva rather than direct excretion from the lacrimal gland (Baca et al., 2007; Lane et al., 2006; van Haeringen & Glasius, 1977). In addition, there is a barrier in the epithelium of the cornea and conjunctiva that prevents glucose from moving into the tear fluid (van Haeringen & Glasius, 1977). In a previous study on cats, the median TG concentration was 13% of the BG concentration, while the median tear urea nitrogen concentration was 109% of that measured in blood plasma (Steinmetz et al., 2019). Similarly, previous studies in humans found very low TG levels in diabetic patients, while the concentration of urea nitrogen in the tears was within the normal range in serum (Kang et al., 1988; van Haeringen & Glasius, 1977). The results of the present study still demonstrated barrier functions against glucose, showing that the values of TG were significantly lower than those of BG. Only 7.52% of the average BG concentration was measured in tears in the present study.

Owing to these two reasons, including the origin of tears and barriers in the epitheliums of the cornea and conjunctiva, the concentration of TG is closely related to the methods of tear collection (Baca et al., 2007; van Haeringen & Glasius, 1977). There are several methods for collecting tears, including the use of STT strips, sponges, or microcapillary tubes. Among them, mechanically stimulating methods may irritate or injure the conjunctiva, resulting in elevated TG levels. This is attributed to leakage of glucose from conjunctival epithelial cells or from extracellular tissue fluid into tear fluid (Baca et al., 2007; van Haeringen & Glasius, 1977). In humans, the highest TG concentrations were found in studies that used mechanically irritative STT strips to extract tear fluid (Baca et al., 2007). This also explains why studies measuring TG in humans showed quite varying concentrations (Baca et al., 2007; Zhang et al., 2011). Unaltered tear samples can be acquired, and reflex tearing can be avoided by using microcapillary tubes because tears are collected directly from the inferior lacrimal lake by capillary action (Sebbag & Mochel, 2020). Additionally, the binding of tear compounds to the collecting device can be minimised (Sebbag & Mochel, 2020). Therefore, in the current study, we used the least irritating method via a microcapillary tube to avoid microtrauma during tear collection and to obtain the most accurate values.

According to our results, the lag time between BG and TG concentration was 10 min (Figure 3a). The lag time in the present study was defined as the time difference between the highest BG level and the highest TG level after glucose injection. TG tracked BG with a 10‐min delay. A lag time of approximately 10 min has also been reported in earlier studies in humans and rabbits (Andreea et al., 2012; Chu et al., 2011; Iguchi et al., 2007; La Belle et al., 2016). However, these lag times varied from 5 to 20 min depending on the experimental designs and measurement methods (Baca et al., 2007; Geelhoed‐Duijvestijn et al., 2021; LeBlanc et al., 2005). The lag time would be affected by both physiological and mechanical lag times in sample collection (Stout et al., 2001). For TG, the physiological lag time reflects the dispersion from plasma to extracellular tissue fluid, such as ISF, and then from the extracellular compartment to tears. Therefore, it is understandable that the 10‐min delay of TG in the present study was like the lag time between ISF and plasma in dogs, which has been reported to be 5–12 min considering the source of TG (Rebrin & Steil, 2000).

Interestingly, considering tear dynamics, the delay time of approximately 10 min for TG equilibration was found to be similar to the time required for the tear film to fully replenish in a dog (approximately 8.20 min) (Sebbag et al., 2019). Further studies are needed to investigate whether other tear metabolites have identical delay times against BG under the influence of the tear turnover rate.

The lag time between TG and BG concentrations is clinically important to determine whether tears can be used as an alternative to invasive blood collection for glucose measurement in dogs with suspected DM. Although tears do not reflect BG in real time, the delay time of 10 min is short. In addition, according to the results of this experiment, which monitored TG and BG every 30 min, TG could reflect the changes in BG. Therefore, the lag time between TG and BG can be considered negligible for evaluating the usual BG levels in clinics.

However, the lag time may not always be constant. In the case of ISF in humans, there was a variation in lag times at the points of rise, fall, and nadir of BG (Boyne et al., 2003). The lag phase was more inapparent or shorter during a fall than a rise in BG levels (Boyne et al., 2003; Sternberg et al., 1996). As TG is mainly derived from ISF, it could be expected that the lag of TG has similar characteristics to the lag of ISF. However, in the current study, no apparent difference in the lag time of tears between the rise and fall in BG was observed.

In the present study, the average TG concentration during hyperglycaemia was significantly higher than that during normoglycaemia. Additionally, the diagnostic accuracy (sensitivity and specificity) signified that hyperglycaemia could be detected via TG concentration. These findings suggest that tears can be used as an early screening tool for DM in dogs. Furthermore, tears have advantages over other body fluids such as ISF, saliva, sweat, and urine, for potential clinical utility. First, tears can be obtained in a quick, simple, and non‐invasive manner. Tear fluid is continuously replenished while maintaining a relatively constant volume (Peng et al., 2013). Since the tear volume in dogs is at least five times greater than the tear volume in humans, the tear meniscus is more easily accessible (Sebbag et al., 2019). Second, a tear sample is not as diluted or contaminated as saliva or urine (Peng et al., 2013). Finally, early detection of hyperglycaemia in tears is possible before glycosuria develops. Glucose is not detected in the urine until the BG concentration exceeds the renal tubular capacity for glucose reabsorption, which occurs during extreme hyperglycaemia (Ettinger et al., 2017). By contrast, based on the results of this study, tears can be used to detect hyperglycaemia without clinical symptoms that does not exceed the maximum renal glucose resorption threshold. Therefore, TG level measurement could be used as a good screening test for DM in dogs. However, to be used clinically as a routine screening test in more patients, a sample collection system that makes it easier to collect tears and shows the BG levels should be devised.

Additionally, the finding of a higher TG level with a higher BG level could guide future research on TG as one of the tear metabolites. For example, research on ocular changes caused by elevated TG levels may help understand various pathological changes in the corneas and conjunctivas of diabetic patients.

There were several limitations to the present study. First, this study used clinically healthy dogs without DM. We compared TG levels with BG levels by inducing transient hyperglycaemic changes in healthy dogs through intravenous glucose administration. Additionally, this study conducted repeated tests, which did not consider differences based on breed, age, and gender. Despite these limitations, we were able to quantify TG levels during normoglycaemia and hyperglycaemia and the cross‐correlation between changes of BG and TG levels was found. However, diabetic patients are considered to be different from normal dogs because BG is maintained at a high concentration most of the time. The current degree of glycaemic control or administration of exogenous insulin can also affect TG concentrations and lag time. Therefore, further studies are required using subjects of different breed, age, and gender or using diabetic dogs to measure TG levels and compare those values to BG levels. Second, the volume (10 μl) of tears we obtained was more than that used in prior studies that measured TG levels in rabbits (Peng et al., 2013) and humans (Cha et al., 2014). During tear collection, decreased tear elimination could alter the TG level (Baca et al., 2007). Since canine median tear volume (Sebbag et al., 2019) (65.3 μl) is much higher than that of rabbits (Chrai et al., 1973) (7.5 μl) and humans (Mishima et al., 1966) (7.0 μl), dogs may be less affected by reflex tearing. However, using a lower volume of tear fluid could reduce the risk of acquiring stimulated tear and would yield more reliable values of the actual reference range under both normal and hyperglycaemic conditions in dogs. Thus, a measurement method that can analyse TG in a low volume is recommended. In human studies, reported TG concentrations were generally lower, as the analytical methods required less sample volume (Baca et al., 2007). Further studies on the use of measurement techniques that require lower tear volumes for TG detection in dogs are needed to determine the most accurate value of basal tears. For example, in previous studies using 1‐μl tear samples in humans, TG was quantified using electrospray ionisation mass spectrometry (Baca et al., 2007; Taormina et al., 2007) or by measuring output current from an electrochemical blood glucose glucometer strip (Cha et al., 2014). In addition, the difference in the measurement method of BG and tears may also be considered a limitation. Although the results of the glucometer (Accu‐Check^®^ Active) were generally higher than the corresponding results of the glucose colorimetric assay in our pilot experiment, we chose the glucometer for monitoring BG concentration because it is the most commonly used device in general practice.

In conclusion, this study quantitatively measured the concentration of TG in dogs, which could provide preliminary data for future research on TG analysis. Furthermore, the TG concentration had a good correlation with that of BG. Considering the significant elevation of TG concentration during hyperglycaemia compared to that during normoglycaemia, tear collection, even at home by owners, can be a potential alternative to invasive blood collection as a screening tool for DM in dogs.

## CONFLICT OF INTEREST

The research was sponsored by URIVET KOREA Co., Ltd., Korea.

## AUTHOR CONTRIBUTIONS

E.L. conceptualised the idea of the study, performed investigation, and wrote the original draft. J.S., D.J., Y.J., and J.A. performed investigation and validation. S.K. and K.S. performed supervision and reviewed and edited the manuscript.

## ETHICS

The authors confirm that the ethical policies of the journal, as noted on the journal's author guidelines page, have been adhered to and the appropriate ethical review committee approval has been received. This study was approved by the Institutional Animal Care and Use Committee of the Seoul National University (SNU‐190930‐5‐3).

### PEER REVIEW

The peer review history for this article is available at https://publons.com/publon/10.1002/vms3.788.

## Data Availability

Research data are not shared.
